# The impact of an extreme climatic disturbance and different fertilization treatments on plant development, phenology, and yield of two cultivar groups of *Solanum betaceum* Cav.

**DOI:** 10.1371/journal.pone.0190316

**Published:** 2017-12-29

**Authors:** Joffre V. Tandazo-Yunga, Mario X. Ruiz-González, Jacqueline R. Rojas, Edwin D. Capa-Mora, Jaime Prohens, José D. Alejandro, Pablo G. Acosta-Quezada

**Affiliations:** 1 Departamento de Ciencias Biológicas, Universidad Técnica Particular de Loja, San Cayetano Alto s/n, Loja, Ecuador; 2 Proyecto Prometeo SENESCYT—Universidad Técnica Particular de Loja—UTPL, Departamento de CC. Biológicas, San Cayetano Alto s/n, Loja, Ecuador; 3 Instituto de Conservación y Mejora de la Agrodiversidad Valenciana, Universitat Politècnica de València, Camino de Vera 14, Valencia, Spain; Karnatak University, INDIA

## Abstract

Changing climatic conditions impose a challenge both to biodiversity and food security. The effects of climate change affect different aspects of the plant or crop, such as morphological and phenological aspects, as well as yield. The effects of greenhouse conditions might be comparable in some cases to a permanent extreme disturbance in climate and weather, thus, contributing to our knowledge on climate change impacts on plant species. We have investigated the differences for 23 traits in two cultivar groups of an Andean traditional crop, *Solanum betaceum*, under two different environmental conditions that correspond to the traditional practices in the open field and three cultural managements under greenhouse conditions (no fertilization or control, organic, and mineral). We found that traditional practices in the open field are the less productive. Moreover, in warmer and drier conditions the treatment with organic fertilization was the most productive. Greenhouse conditions, however, delay production. We further identified traits that differentiate both cultivar groups and traits that are linked to either the new climate conditions or the fertilization treatments. Fruit characteristics were quite homogeneous between the two cultivar groups. Overall, our results provide insight on the consequences that climate change effects might exert on crops such as tree tomato, reveal that greenhouses can be a robust alternative for tree tomato production, and highlight the need to understand how different managements are linked to different solutions to fulfil the farmers’ demands.

## Introduction

Plant growth and development depend on species-specific temperature ranges. Among the expected climate change effects, the rising of temperatures might have dramatic consequences on biodiversity and plant yield [1]. Thus, although we might expect enhanced plant growth, higher temperatures may cause yield reduction [2]. Indeed, changes in temperature induce stress in plant tissues affecting the reproductive stage by delaying or accelerating flowering, affecting differently male and female structures or inducing defects and deformities on the reproductive structures [3]. That is, the effects of climate change have a direct impact on plant biodiversity and may accelerate extinction events or facilitate invasions [4, 5] which also affect many domesticated species with economical and nutritional value. Therefore, the consequences are so serious that research on food security, sustainable agriculture, conservation of biodiversity, and the development of strategies to build up resilience against climate change effects have become a priority in many national and international political agendas (e.g. UE, H2020).

Traditional cultural practices of local farmers face many different challenges and changing climatic conditions is one of them. In this respect, the enhancement of emerging crops from developing countries might be greatly affected by climate change. Moreover, the import demand of fresh tropical fruit in developed countries has increased by more than 70%, which represents an important income source for producers in developing countries [6]. Thus, research on the fate of plants in general and crops in particular when facing extreme climate disturbances is fundamental to develop new policies and strategies to strengthen food security. To contribute to this issue we have selected a traditional Andean crop species, the tree tomato or tamarillo.

Tree tomato or tamarillo (*Solanum betaceum* Cav.) is a fast-growing small tree native to South America that produces edible fleshy fruit with a growing market in its native region in Andean countries, as well as in Europe, North America, and Oceania [7, 8]. The fruit have a high content in ascorbic acid, pro-vitamin A, carotenoids, and vitamin B6, as well as a high antioxidant activity [9–11]. Three main morphological types were conventionally recognized based on fruit colour: dark red or purple, red-orange, and yellow [12]. Recent research has established five cultivar groups based on fruit colour and shape: orange, orange pointed, purple, red, and red conical [13]. Moreover, there are large differences among cultivars for important traits relevant for fruit flavour, like soluble sugars and organic acids [10, 14]. Morphological and molecular studies on cultivars from Ecuador, Bolivia, Peru, and Colombia, however, have unveiled the existence of great genetic diversity in the species [13, 15]. The region of origin where tree tomato has evolved under cultivation in its region of origin has less intra-annual climatic variability than other regions of the world, albeit the existence of many microclimates has led to the existence of an ecological mosaic that Andean farmers have learnt to take advantage of since the time of the Incas [16], and which might partially explain the genetic diversity found among cultivars.

In general, the tree tomato plant reaches a height of one to five meters, with a lifespan of five to 12 years [7]. The plant has a woody stem that branches out above the 1.5 m and forms a wide crown. Their long-lived leaves are large (up to 40 × 30 cm) with strong fragrance. The inflorescences are terminal scorpioid cymes, and its flowers, functional for 5 days and in numbers of up to 50 per inflorescence, are clustered and produce one to six egg-sized fruits [17]. The plants start to produce fruit after their first year of life. While tree tomato is mostly autogamous and self-compatible, the species benefits from the help of either wind or pollinators for fecundation [18]. As there is broad morphological heterogeneity among tree tomato cultivar groups, several quantitative morphological traits, like those related to fruit and infructescence, show high heritability and are good descriptors to differentiate among cultivar groups [13].

Tree tomato seems to have good adaptability potential to different environmental conditions [7] but to our knowledge no studies have explored the effects of climate, fertilization, and their interaction in this crop. In fact, crop adaptability to changing conditions is pivotal to increase crop diversity and to cope with changing agrosystems potentially endangered by climate change [19], thus contributing to food security [20]. An important tool that allows the thoughtful study of the latter effects on a single species is the existence of properly defined descriptors that characterize its agromorphology (e.g., Bioversity International descriptors), biology, and phenology in different environmental conditions (e.g., BBCH descriptors). In the case of tree tomato both types of descriptors are available [21, 22]. Here we address two main questions: 1) how does an extreme climate disturbance affect *S*. *betaceum* biology when compared to its traditional conditions? And 2) Does different fertilization treatments improve yield under the new environmental conditions? In order to investigate the role of both climate change and fertilization on tree tomato architecture, phenology, and yield, as well as to provide insight on potential new production alternatives for this emerging crop we set up four treatments, the traditional crop culture of tree tomato (open-field) and three controlled greenhouse conditions (no fertilization, organic, and mineral fertilization).

## Material and methods

### Location, plant material, and cultivation

Experiments were initiated on September 14^th^, 2014 and lasted until the end of January 2016 at a site located in the Universidad Técnica Particular de Loja (UTPL, Loja, Ecuador) with coordinates 4° 0’ 1.59”S and 79° 10’ 48.46”W. The site has an altitude of 2160 m a.s.l., an average minimum/maximum temperature across the year of 12.9°C and 22.6°C (mean ± SE), an average annual precipitation and a relative humidity of 780 mm and 83%, respectively. In the greenhouse, we recorded a diurnal maximum temperature variation between 28.8°C and 35.5°C and relative humidity of 55%. The site corresponds to the low dry montane forest (bs-MB) formation [23]. We selected seeds from two cultivar groups of tree tomato, orange pointed and purple. We chose these two cultivar groups because they are representative of the species morphological diversity, are the most productive ones, and are promising as a tree tomato alternative product market, as found previously [13]. The seeds were sown in nursery bags in a 3:2:1 mixture of organic soil:sand:earthworm humus. Then, seedlings were transplanted to open field and greenhouse plots after 60–70 days post-germination into 0.5 × 0.5 m holes. Distance among plants was at 2 × 2 m. The latter was thought to compare the standard local farmers’ conditions with both a more developed management and to test the effects of climate change on the crop. Using field conditions as the farmers would normally do are a guiding principle for an on-farm trial [24]. Moreover, for some trials involving certain descriptors is opportune to set up them under controlled conditions, such as in a greenhouse [20].

### Experimental design

Plants of the two cultivar groups were grown alternatively in random blocks under four different treatments; the first three treatments (40 plants per cultivar group) were kept in greenhouse and the fourth in open field conditions (15 plants per cultivar group). Treatment 1 consisted in organic fertilization based on humic acids 20.2% (humic acid 10%, fulvic acid 10.2%, K_2_O 3.2%) at a concentration of 0.4 L ha^-1^ every fortnight. In treatment 2 we applied a mineral fertilization based on 10:10:40 during plant development and 20:20:20 during flowering and fructification, at a concentration of 25–9.58–20.8 kg N-P_2_O_5_-K_2_O ha^-1^ every two weeks. Treatment 3 was a control with no fertilization, and treatment 4 simulated traditional crop practices (open-field) with a basal N-P_2_O_5_-K_2_O 12-36-12 fertilization every two months up to 120 kg ha^-1^ and year. All fertilization treatments were designed after a soil analysis in the soil laboratory at UTPL. Traditional cultural practices in the open field do not consider irrigation due to weather conditions in Andean communities. In greenhouse conditions we installed a drop irrigation system as a ring to irrigate the plants three times per week and, thus, avoid hydric stress.

### Morphoagronomic characterization

The evaluation of plant development and production was performed using 14 descriptors; ten of them are based on those published by Bioversity International [21], to evaluate plant architecture, inflorescence and fruit characters, and yield (Table 1): stem length (7.1.2), stem diameter (7.1.3), crown maximum radius (7.1.5), number of main branches (7.1.6), number of fruit (7.4.1), fruit length (7.4.9), fruit width (7.4.10), fruit pedicel length (7.4.12), fruit apex angle (7.4.8), and fruit weight (7.4.14). In addition, we measured the number of nodes and inflorescences and calculated a fruit shape factor as length / width ratio and the yield (overall weight of fruit produced per plant). For most descriptors, measures were registered directly in the field and other characters were evaluated in the laboratory by using image-processing tools, like the UTHSCSA Image Tool (University of Texas Health Science Center, San Antonio, Texas, USA) software, using scanned images.

**Table 1:** Agronomic traits for plant architecture, fruit characters and yield, and phenology of *S*. *betaceum*.

Evaluated variables	Code	Instructions for measurement and description
**I. Plant architecture**		
Stem length (cm)	7.1.2	Distance from the stem base to the first branching point.
Stem diameter (cm)	7.1.3	Stem diameter at 30 cm below the first branching point.
Crown maximum radius (cm)	7.1.5	Diameter of the horizontal projection on the ground of the crown.
Number of nodes	-	Number of nodes, equivalent to the number of leaves, present in the stem after 6 months
Number of main branches	7.1.6	Branching pattern in the central stem.
**II. Fruit characteristics and yield**		
Fruit length (cm)	7.4.9	Length of the berry from the proximal to the distal part.
Fruit width (cm)	7.4.10	Maximum width of the berry
Fruit apex angle (degrees)	7.4.8	Angle formed by the berry edges at 2 cm of the apex
Fruit shape factor	-	Ratio between fruit length (7.4.9) and fruit width (7.4.10)
Fruit pedicel length (cm)	7.4.12	Length of the fruit pedicel.
Inflorescences number	-	Number of inflorescences produced by the shrub
Number of fruit	7.4.1	Overall number of fruit produced by each adult plant of the same age
Fruit weight (g)	7.4.14	Overall fruit weight / number of fruit produced per plant
Yield (g)	-	Total fruit weight produced by a plant
**III. Phenology (all units in days)**		
30% of final length of stem	303	Days to reach the 30% of the final length of the stem
1^st^ primary apical side shoot	201	Days to see the 1^st^ primary apical side shoot visible
15^th^ leaf on stem unfolded	115a	15^th^ leaf on stem unfolded
25 leaves on the crown	125b	25 or more leaves on the crown unfolded
1st inflorescence visible	501	1^st^ inflorescence visible
1^st^ flower open	601	1^st^ inflorescence with first flower open
10% fruit ripe colour	801	10% of fruit show typical fully ripe colour
50% fruit ripe colour	805	50% of fruit show typical fully ripe colour
90% fruit ripe colour	809	90% of fruit show typical fully ripe colour

Codes denote the Bioversity International [21] or BBCH codifications [22], when applicable.

#### Plant architecture

For plant architecture traits we studied: stem length (7.1.2), stem diameter (7.1.3), crown maximum radius (7.1.5), the number of main branches (7.1.6), and the number of nodes (Table 1). All variables were measured every fortnight. In order to fit to greenhouse requirements, all plants indoors were pruned at around 120 cm, while plants in the open field were not pruned, following traditional handling. We quantified the number of main branches and analysed for differences between cultivar groups and among treatments with a multivariate ANOVA. Overall results are reported as the Pillai’s trace statistic, which is the most conservative *F*-statistic within MANOVA analyses. All statistical analyses were conducted in IBM® SPSS® Statistics v. 24.

### Fruit characteristics and yield

The fruit characteristics studied included fruit length (7.4.9), width (7.4.10), the apex angle (7.4.8), the shape factor, and the pedicel length (7.4.12) to investigate fruit characteristics (Table 1). For yield traits we quantified the inflorescence and fruit (7.4.1) number, the weight of the fruit (7.4.14), and the yield as the overall weight of fruit produced per plant (Table 1). We took five fruits at random from each tree to measure fruit characteristics. We log transformed the number of inflorescences and fruit, and the overall weight of fruit per plant. Both data sets were analysed with a multivariate ANOVA. Overall results are reported as the Pillai’s trace statistic and post hoc comparisons using the conservative Scheffé means comparison test were conducted for the treatment factor. Several trees died or did not produce fruit so we tested for differences in the number of trees alive among cultivar groups and treatments with a *χ*^2^ test [25].

### Phenology

We analysed for differences in nine variables based on the tree tomato extended BBCH scale [22], which included the days needed to: 1) reach the 30% of the final length of the stem (303); 2) first primary apical side shoot being visible (201); 3) 15^th^ leaf on the stem being unfolded (115a); 4) 25 or more leaves on the crown being unfolded (125b); 5) first inflorescence being visible (501); 6) first inflorescence with the first flower being opened (601); and 7–9) 10%, 50%, and 90% of the fruit showing the typical fully ripe colour (801, 805, and 809, respectively; Table 1). The first four traits are related with vegetative development and architecture, while the last five describe the formation of reproductive structures and the production of mature fruit. Variables 303, 201, 601, and 801 were Box-Cox transformed to meet the assumptions of the ANOVA. We conducted a multivariate ANOVA. Overall results are reported as the Pillai’s trace statistic and post hoc analyses using Scheffé means comparison test.

## Results

### Plant architecture

A summary describing all plant architecture studied traits for each tree tomato cultivar group and treatment is provided in Table 2. Orange pointed cultivar group tree tomato shrubs showed overall larger stem lengths (141.14 cm) than the purple ones (136.60 cm). We found an overall treatment difference of 15.85% of stem length between trees cultivated using traditional practices in the open field (156.45 cm) and the tree tomatoes in the greenhouse, which were shorter. Stem diameter, however, was 10.82% wider in the organic fertilization treatment within the greenhouse (5.08 cm) than in the open field (4.53 cm). Crown maximum radius was nearly 50% larger in greenhouse conditions than in the open field, being the largest in the mineral fertilization treatment (247.69 cm) with 7.5% of difference when compared to the organic fertilization (229.16 cm). The plants cultured under the traditional practice showed the highest number of leaves (28.63) produced during the early stages of the plants (number of nodes), nearly 28% more than the plants produced under greenhouse conditions. This difference was statistically significant for treatment but not for variety or their interaction (*F*_3,71_ = 31.493, *p*–value < 0.001; *F*_1, 71_ = 0.646, *p*–value = 0.424; *F*_3, 71_ = 0.379, *p*–value = 0.769; respectively), and was supported by post hoc analysis after Scheffé (*p*–value < 0.001).

**Table 2:** Plant architecture at the end of the first year of production.

Character	Stem length (cm)	Stem diameter (cm)	Crown max radius (cm)	# Nodes
ORANGE POINTED CULTIVAR GROUP				
Greenhouse—Organic				
n	10	10	10	14
Mean	139.20	5.13	223.10	20.57
Min	122.00	4.24	193.00	19.00
Max	159.00	5.98	285.00	23.00
Range	37.00	1.74	92.00	4.00
Max/Min	1.30	1.41	1.48	1.21
SD	10.41	0.69	26.57	1.22
Greenhouse–Mineral				
n	13	13	13	12
Mean	135.54	4.68	252.23	20.75
Min	124.00	3.93	214.00	19.00
Max	147.00	5.57	295.00	25.00
Range	23.00	1.64	81.00	6.00
Max/Min	1.19	1.42	1.38	1.32
SD	7.16	0.44	27.33	2.05
Greenhouse–Control				
n	7	7	7	10
Mean	134.43	4.78	227.29	21.50
Min	125.00	3.27	195.00	18.00
Max	141.00	5.93	264.00	26.00
Range	16.00	2.66	69.00	8.00
Max/Min	1.13	1.82	1.35	1.44
SD	6.78	0.86	27.83	2.51
Field				
n	5	5	5	4
Mean	155.40	4.37	123.60	29.25
Min	149.00	3.90	94.00	26.00
Max	168.00	5.26	187.00	32.00
Range	19.00	1.36	93.00	6.00
Max/Min	1.13	1.35	1.99	1.23
SD	7.70	0.65	36.76	2.50
PURPLE CULTIVAR GROUP				
Greenhouse–Organic				
n	9	9	9	15
Mean	130.78	5.02	235.22	20.93
Min	118.00	3.90	195.00	15.00
Max	139.00	6.06	282.00	25.00
Range	21.00	2.16	87.00	10.00
Max/Min	1.18	1.55	1.45	1.67
SD	6.94	0.66	28.09	2.63
Greenhouse–Mineral				
n	14	14	14	12
Mean	129.21	4.59	243.14	20.50
Min	115.00	3.60	198.00	17.00
Max	140.00	5.50	276.00	25.00
Range	25.00	1.90	78.00	8.00
Max/Min	1.22	1.53	1.39	1.47
SD	7.20	0.54	24.31	2.39
Greenhouse–Control				
n	9	9	9	8
Mean	128.89	4.95	221.89	20.88
Min	120.00	4.06	145.00	18.00
Max	159.00	5.71	279.00	24.00
Range	39.00	1.65	134.00	6.00
Max/Min	1.33	1.41	1.92	1.33
SD	11.75	0.58	40.60	1.89
Field				
n	6	6	6	4
Mean	157.50	4.68	125.00	28.00
Min	130.00	3.94	113.00	26.00
Max	175.00	5.68	137.00	29.00
Range	45.00	1.73	24.00	3.00
Max/Min	1.35	1.44	1.21	1.12
SD	16.53	0.69	9.49	1.41

Open field plants were not pruned after following traditional management of this crop.

After the first year of production there were overall significant effects for cultivar group, treatment, and their interaction (*F*_3,70_ = 2.803, *p*–value = 0.046; *F*_9, 216_ = 11.276, *p*–value < 0.001; *F*_9, 216_ = 2.213, *p*–value = 0.022; respectively). We found differences in stem length for cultivar group, treatment, and their interaction (Table 3). In addition, stem diameter and the maximum radius of the crown showed significant differences for treatment (Table 3). Tree tomato shrubs did produce either two or three main branches. There were significant differences between treatments in the number of main branches produced, being three the branches of the tree tomato shrubs cultured in the open field conditions and two in greenhouse conditions (*F*_3, 63_ = 3.799, *p*–value < 0.001), but not for cultivar group (*F*_1, 63_ = 0.142, *p*–value = 0.294) or their interaction (*F*_3, 63_ = 0.106, *p*–value = 0.481).

**Table 3:** Multivariate analysis for differences between cultivar groups and among treatments for the architecture traits after the first year of production.

	Dependent variable	MS	*F*-value	D.F.	*p*-value
Cultivar group	**Stem length**	**709.085**	**6.175**	**1, 72**	**0.015**
	Stem diameter	34.711	2.249	1, 72	0.138
	Maximum radius of the crown	763.772	1.730	1, 72	0.193
Treatment	**Stem length**	**1218.196**	**10.609**	**3**	**< 0.001**
	**Stem diameter**	**105.180**	**6.814**	**3, 72**	**< 0.001**
	**Maximum radius of the crown**	**30011.956**	**67.976**	**3, 72**	**< 0.001**
Cultivar group × treatment	**Stem length**	**472.959**	**4.119**	**7**	**0.009**
	Stem diameter	37.405	2.423	3, 72	0.073
	Maximum radius of the crown	371.040	0.840	3, 72	0.476

Values in bold denote significant differences.

### Fruit characteristics and yield

We have summarised the information on the studied variables in Table 4. We found that orange pointed cultivar group exhibits larger values for fruit length and diameter (6.49 and 5.03 cm, respectively) and lower values for pedicel length (4.65 cm) when compared to purple cultivar group (6.31, 4.90, and 4.97 cm, respectively). The fruit of open field treatment generally had a higher fruit length/width ratio and therefore were more elongated. Consequently, fruit angle was 5.30% sharper in open field conditions than in the greenhouse and the differences in shape factor (7.75%) indicate rounder fruit in greenhouse conditions (1.25, 1.27, and 1.30 in organic, mineral, and control, vs 1.36 in the open field). Pedicel length had the smallest variation among treatments (1.85%). There were not differences among treatments for the number of trees that fructified (*χ*^2^ = 9.5; d.f. = 7; *p*–value = 0.219).

**Table 4:** Fruit characteristics and yield.

Character	Fruit length (cm)	Fruit diameter (cm)	Apex angle (°)	Shape Factor	Pedicel length (cm)	# Inflorescences	# Fruit	Fruit weight (g)	Yield (g)
ORANGE POINTED CULTIVAR GROUP									
Greenhouse—Organic									
n	44	44	44	44	44	10	10	10	10
Mean	6.49	5.17	91.08	1.26	4.6	42.6	32.8	74.49	2601.3
Min	4.96	4.22	82.54	1.09	3.56	14	13	21.54	280.03
Max	7.68	6.19	98.79	1.52	6	78	64	88.36	5564.58
Range	2.72	1.97	16.25	0.43	2.44	64	51	66.82	5284.55
Max/Min	1.55	1.47	1.2	1.39	1.69	5.57	4.92	4.1	19.87
SD	0.55	0.41	3.57	0.08	0.71	18.37	18.52	19.43	1720.36
Greenhouse–Mineral									
n	61		61	61	61	13	13	13	13
Mean	6.59	5.10	92.04	1.3	4.74	45.15	26.23	71.87	1917.98
Min	5.82	4.31	85.02	1.08	3.56	7	1	33.95	33.95
Max	7.88	5.95	102.77	1.42	6	87	58	84.5	4124.43
Range	2.06	1.64	17.75	0.33	2.44	80	57	50.55	4090.48
Max/Min	1.35	1.38	1.21	1.31	1.69	12.43	58	2.49	121.49
SD	0.35	0.36	3.58	0.07	0.48	22.59	15.66	12.91	1058.27
Greenhouse–Control									
n	39		38	38	39	7	7	7	7
Mean	6.47	5.03	90.75	1.29	4.69	43.5	16.57	78.14	1270.08
Min	5.21	4.06	80.78	1.05	2.5	8	3	68.06	204.18
Max	7.48	6	100.37	1.5	6.64	76	33	88.41	2516.2
Range	2.27	1.94	19.59	0.45	4.14	68	30	20.35	2312.02
Max/Min	1.44	1.48	1.24	1.43	2.66	9.5	11	1.3	12.32
SD	0.54	0.34	4.35	0.11	0.76	26.98	11.93	9.1	871.31
Field									
n	14		14	14	14	5	5	5	5
Mean	6.41	4.80	86.75	1.34	4.57	22.6	9	74.28	673.72
Min	5.9	4.39	78.87	1.1	3.62	17	3	66.5	199.51
Max	6.92	5.52	97.85	1.51	5.89	39	18	79.78	1358.47
Range	1.02	1.13	18.98	0.41	2.27	22	15	13.27	1158.96
Max/Min	1.17	1.26	1.24	1.37	1.63	2.29	6	1.2	6.81
SD	0.31	0.32	5.19	0.11	0.67	9.21	6	5.12	451.02
PURPLE CULTIVAR GROUP									
Greenhouse–Organic									
n	36		36	36	36	9	9	9	9
Mean	6.23	5.05	92.14	1.24	4.95	51.78	25.67	77.03	1992.25
Min	5.21	3.9	81.33	1.1	2.8	27	11	66.02	726.22
Max	7.14	5.85	101.77	1.51	6.54	104	43	90.61	3691.68
Range	1.93	1.95	20.44	0.41	3.74	77	32	24.59	2965.46
Max/Min	1.37	1.5	1.25	1.37	2.34	3.85	3.91	1.37	5.08
SD	0.51	0.45	4.33	0.1	0.77	25.61	11.28	8.07	921.37
Greenhouse–Mineral									
n	66		64	64	66	14	14	14	14
Mean	6.17	4.97	91.1	1.24	4.99	46.15	17.5	74.94	1309.95
Min	5.2	4.25	84.87	1.05	3.22	33	1	48.95	66.46
Max	7.16	5.9	102.84	1.38	6.5	72	38	100.4	3574.8
Range	1.96	1.65	17.97	0.32	3.28	39	37	51.45	3508.34
Max/Min	1.38	1.39	1.21	1.31	2.02	2.18	38	2.05	53.79
SD	0.45	0.38	3.89	0.07	0.61	10.38	10.23	14.45	898.57
Greenhouse–Control									
n	18		18	18	18	8	8	8	8
Mean	6.25	4.79	89.32	1.31	4.91	32.25	33.88	74.06	2218.94
Min	5.53	4	82.18	1.12	4.2	20	9	46.13	680.31
Max	7.07	5.8	97.23	1.47	6.27	47	93	93.71	5493.81
Range	1.54	1.8	15.05	0.35	2.07	27	84	47.58	4813.5
Max/Min	1.28	1.45	1.18	1.31	1.49	2.35	10.33	2.03	8.08
SD	0.41	0.48	4.08	0.08	0.66	13.79	29.43	14.84	1554.32
Field									
n	25		25	25	25	6	6	6	6
Mean	6.58	4.82	86.76	1.37	5.02	14.83	8	83.58	657.86
Min	5.74	4.17	77.3	1.23	3.54	6	1	60.49	95.71
Max	7.45	5.82	95.3	1.62	6.2	25	13	99.75	1281.16
Range	1.71	1.65	18	0.39	2.66	19	12	39.25	1185.45
Max/Min	1.3	1.40	1.23	1.32	1.75	4.17	13	1.65	13.39
SD	0.45	0.49	4.19	0.09	0.61	7.78	4.82	16.61	432.07

Summary of each of the nine studied traits.

We found overall effects for cultivar group, treatment, and their interaction, when analysing the five variables related to yield (*F*_5, 288_ = 6.644, *p*–value < 0.001; *F*_15, 870_ = 4.624, *p*–value < 0.001; and *F*_15, 870_ = 1.942, *p*–value = 0.017, respectively). Fruit diameter and length, and pedicel length were different between the two cultivar groups (Table 5). Treatment had a significant effect on fruit diameter, fruit apex angle, and the shape factor. When studying the interaction between cultivar group × treatment there were differences for fruit length and the shape factor. Post hoc analysis detected that fruit diameter in the open field management was different from organic and mineral treatments in the greenhouse (*p*–value = 0.002 and 0.030, respectively). The angle of the fruit apex is significantly different in the outer control compared to all other three treatments (≤ 0.001). When considering the fruit shape factor, fruit produced in the field were significantly different compared to greenhouse treatments (organic and mineral, *p*–value < 0.001; greenhouse control, *p*–value = 0.007.

**Table 5:** Multivariate ANOVA for fruit characteristics and yield.

	Dependent variable	MS	D.F.	*F*-value	*p*-value
Fruit characteristics					
Cultivar group	**Fruit diameter**	**0.744**	**1, 292**	**4.662**	**0.032**
	**Fruit length**	**1.932**	**1, 292**	**8.970**	**0.003**
	Fruit apex angle	6.088	1, 292	0.380	0.538
	Shape factor	0.002	1, 292	0.241	0.624
	**Pedicel length**	**5.911**	**1, 292**	**13.854**	**< 0.001**
Treatment	**Fruit diameter**	**0.940**	**3, 292**	**5.889**	**0.001**
	Fruit length	0.171	3, 292	0.795	0.498
	**Fruit apex angle**	**246.546**	**3, 292**	**15.376**	**< 0.001**
	**Shape factor**	**0.109**	**3, 292**	**14.108**	**< 0.001**
	Pedicel length	0.143	3, 292	0.336	0.799
Cultivar group × treatment	Fruit diameter	0.113	3, 292	0.707	0.548
	**Fruit length**	**0.784**	**3, 292**	**3.640**	**0.013**
	Fruit apex angle	21.926	3, 292	1.367	0.253
	**Shape factor**	**0.025**	**3, 292**	**3.210**	**0.023**
	Pedicel length	468.572	3, 292	1.685	0.170
YIELD					
Cultivar group	**Log # inflorescences**	0.001	1, 64	0.017	0.897
	**Log # fruit**	0.005	1, 64	0.035	0.852
	**Average fruit weight**	118.217	1, 64	0.622	0.433
	**Yield**	81191.606	1, 64	0.064	0.800
Treatment	**Log # inflorescences**	**0.745**	**3, 64**	**11.979**	**< 0.001**
	**Log # fruit**	**0.794**	**3, 64**	**5.297**	**0.003**
	**Average fruit weight**	84.450	3, 64	0.445	0.722
	**Yield**	**6202750.117**	**3, 64**	**4.927**	**0.004**
Cultivar group × treatment	**Log # inflorescences**	0.037	3, 64	0.599	0.618
	**Log # fruit**	0.215	3, 64	1.438	0.240
	**Average fruit weight**	96.612	3, 64	0.509	0.678
	**Yield**	2311439.403	3, 64	1.836	0.150

We studied the number of inflorescences and fruit, the weight of the fruit, and the yield for all tree tomato tree. Fruit characteristics were measured on five fruits per shrub. Values in bold are significant.

Regarding yield traits, there were 60.34% less inflorescences (above 50% less outdoors than indoors) and 70.93% less fruit in the open field (8.50) than in the organic fertilization treatment (29.24). The average weight of the fruit, however, was 7% larger in the traditional treatment (78.93 g) than in the greenhouse (73.41–76.10 g). Overall, yield was 71% higher in the organic fertilization treatment (2296.78, 1613.97, and 1744.51 g in organic, mineral, and control, respectively) than in the open field (665.79 g) and 30% higher than yield in the mineral fertilization treatment.

**Fig 1:**
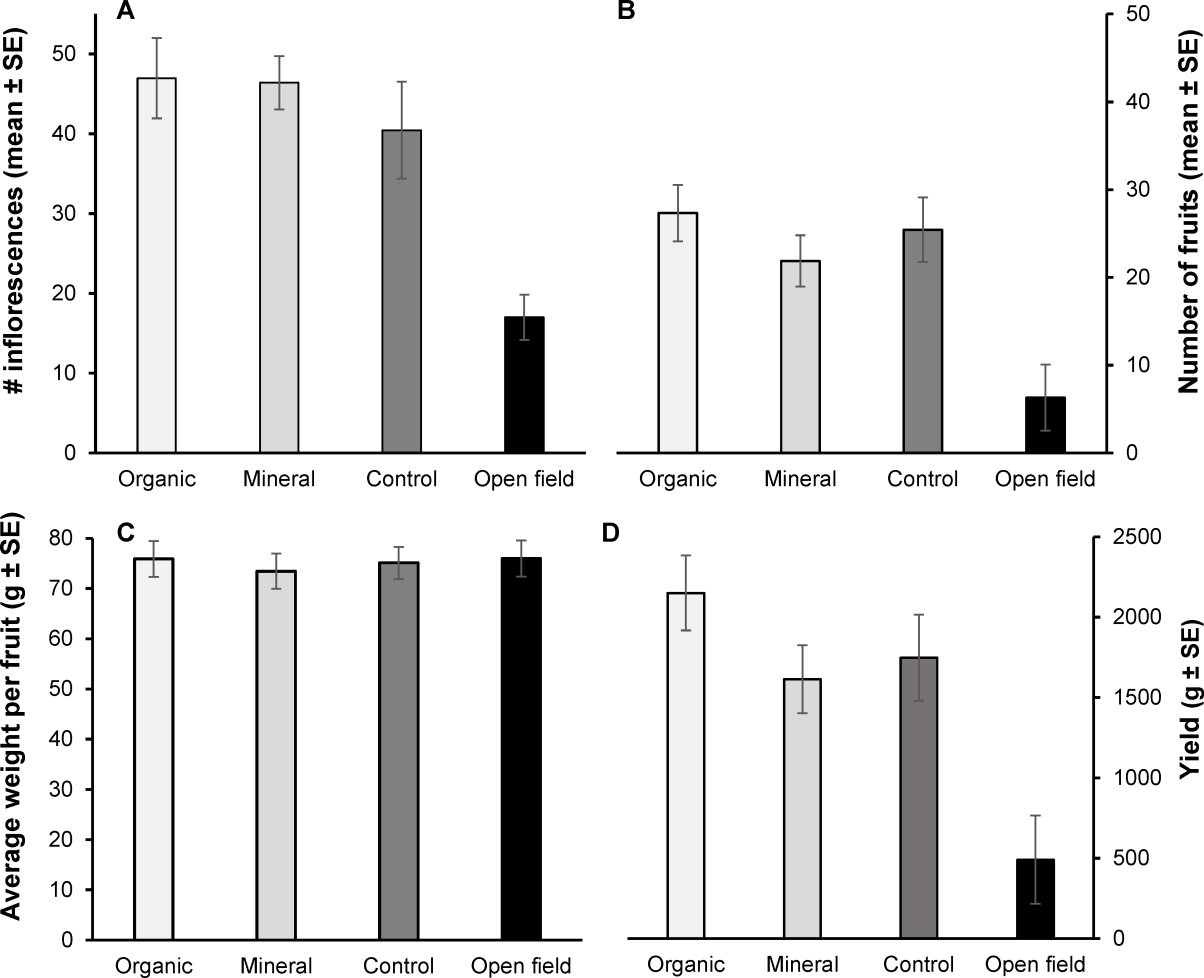
*S*. *betaceum* fruit characteristics and yield traits per treatment. (A) mean number of inflorescences. (B) mean number of fruit. (C) average weight per fruit. (D) yield. Bars denote SE.

We found that for all the four variables analysed for the whole number of tree tomato trees, treatment had overall significant effect (*F*_12, 189_ = 3.353, *p*–value < 0.001). Cultivar group and the interaction cultivar group × treatment, however, had no significant effects (*F*_4,61_ = 0.180, *p*–value = 0.948; *F*_12, 189_ = 1.184, *p* = 0.297; respectively). Open field treatment resulted in a lower number of fruit with larger weight when compared to other treatments (*p*–values < 0.003; Fig 1).

### Phenology

The description of each of the nine variables is provided in Table 6. Plants in the open field reached the 30% of their final length a 22.67% later than plants in the greenhouse, where plants in the organic fertilization treatment were the fastest with 162 days. Plants in the field produced an average number of 30 nodes, which represents the 100% of the final length of the stem [22], thus, we measured the 30% of the final length when nine nodes were present. The maximum time needed to reach that point was between 222 and 225 days in the field (orange pointed and purple cultivar groups, respectively) and the minimum was between 119 and 148 days (orange pointed cultivar group in organic fertilization treatment and purple cultivar group in the mineral fertilization treatment, respectively). Orange pointed cultivar group needed four days less (173.34) than the purple cultivar group (177.00) to produce nine nodes. The same pattern was found when looking for the presence of the first apical shoot, when the development of the crown starts, with 11.69% of delay outdoors. We found five days of difference between the cultivars, being the orange pointed (206.21 days) faster than the purple. The presence of the 15^th^ leaf on the stem unfolded was faster in the mineral fertilization treatment (207.97 days) and 18.23% slower in the open field. The presence of inflorescences and the opening of the first flower, however, happened first in the open field (6.01 days and 11.64% faster) and in orange pointed cultivar group (231.21 days) and was slower in the organic fertilization treatment (14 and 32 days later, respectively). The presence of at least 25 leaves on the crown unfolded was 14.62% faster in the organic fertilization treatment than in the open field (48 days later). The appearance of 10, 50, and 90% of fruit typical fully ripe colour was 5–6 days faster in the orange pointed cultivar group (469.34, 487.05, 508.83 days, respectively) than in the purple cultivar group. Moreover, these three phenological traits appeared faster in the open field (between 18.3 and 20%) than in the greenhouse, where the organic fertilization treatment was faster in all the three characteristics. Thus, the production of 90% of the mature fruit needed between 428 and 436 days (orange pointed and purple cultivar groups, respectively) after the transplant in the open field, while plants in the greenhouse needed between 532–538 and 539–541 days (orange and purple cultivar groups, respectively).

**Table 6:** Descriptors for the first nine studied phenological traits.

Trait Treatment	303	201	115a	125b	501	601	801	805	809
**ORANGE**									
**Greenhouse–Organic**									
n	15	15	15	15	15	15	15	15	15
Mean	158.07	199.00	217.47	277.53	237.33	274.87	476.80	508.87	532.40
Min	119	192	182	265	228	250	466	493	519
Max	210	204	253	296	250	309	492	521	543
Range	91	12	71	31	22	59	26	28	24
Max/Min	1.76	1.06	1.39	1.12	1.10	1.24	1.06	1.06	1.05
SD	33.26	3.61	20.00	10.60	6.80	19.79	7.04	7.98	6.46
**Greenhouse—Mineral**									
n	15	15	15	15	15	15	15	15	15
Mean	163.33	205.33	203.27	278.73	232.60	273.07	499.20	508.93	539.40
Min	144	192	168	243	221	251	473	491	528
Max	228	216	239	327	253	293	523	540	557
Range	84	24	71	84	32	42	50	49	29
Max/Min	1.58	1.13	1.42	1.35	1.14	1.17	1.11	1.10	1.05
SD	23.23	7.53	20.89	18.53	8.69	15.57	11.32	10.47	6.73
**Greenhouse—Control**									
n	10	10	10	10	10	10	10	10	10
Mean	165.9	197.7	226.4	287.2	230.9	269.4	494.9	511.6	535.1
Min	149	189	182	275	212	244	474	502	526
Max	208	218	253	302	240	307	511	521	550
Range	59	29	71	27	28	63	37	19	24
Max/Min	1.40	1.15	1.39	1.10	1.13	1.26	1.08	1.04	1.05
SD	20.92	8.12	21.20	8.65	7.78	20.00	12.61	4.77	8.96
**Field**									
n	15	15	15	15	15	15	15	15	15
Mean	206.07	222.80	251.20	333.53	224.00	242.20	406.47	418.80	428.40
Min	193	201	221	298	205	229	381	401	414
Max	222	237	273	371	242	258	424	434	445
Range	29	36	52	73	37	29	43	33	31
Max/Min	1.15	1.18	1.24	1.24	1.18	1.13	1.11	1.08	1.07
SD	7.52	10.71	16.05	17.80	9.17	7.60	12.71	9.17	8.33
**PURPLE**									
**Greenhouse–Organic**									
n	15	15	15	15	15	15	15	15	15
Mean	165.93	201.53	209.80	284.80	240.60	278.67	492.20	513.33	538.33
Min	150	190	172	270	230	259	479	500	529
Max	182	206	245	298	252	298	499	527	544
Range	32	16	73	28	22	39	20	27	15
Max/Min	1.21	1.08	1.42	1.10	1.10	1.15	1.04	1.05	1.03
SD	12.81	4.44	20.82	8.98	5.45	11.90	5.67	6.94	4.92
**Greenhouse–Mineral**									
n	15	15	15	15	15	15	15	15	15
Mean	162.93	208.00	212.67	292.27	239.33	279.13	495.87	516.87	541.60
Min	150	193	182	277	220	267	480	497	530
Max	182	221	250	324	255	311	505	526	549
Range	32	28	68	47	35	44	25	29	19
Max/Min	1.21	1.15	1.37	1.17	1.16	1.16	1.05	1.06	1.04
SD	9.48	9.04	21.15	15.57	9.95	12.86	6.65	6.97	4.91
**Greenhouse–Control**									
n	10	10	10	10	10	10	10	10	10
Mean	166.2	204.1	221.2	295.8	236.8	277.7	504.7	522.7	537.8
Min	148	198	184	263	223	255	497	499	527
Max	179	219	252	318	248	310	521	538	551
Range	31	21	68	55	25	55	24	39	24
Max/Min	1.21	1.11	1.37	1.21	1.11	1.22	1.05	1.08	1.05
SD	9.52	5.88	24.62	19.09	7.02	20.67	7.90	11.55	7.25
**Field**									
n	15	15	15	15	15	15	15	15	15
Mean	212.93	230.73	257.47	325.07	225.20	246.93	409.73	417.00	435.67
Min	196	196	225	289	202	228	386	398	418
Max	225	241	279	372	237	260	426	429	451
Range	29	45	54	83	35	32	40	31	33
Max/Min	1.15	1.23	1.24	1.29	1.17	1.14	1.10	1.08	1.08
SD	8.41	11.87	16.92	20.37	9.70	7.73	9.53	8.32	8.88

Trait 303: 30% of final length of stem; trait 201: 1st primary apical side shoot; trait 115a, 15th leaf on stem unfolded; trait 125b, 25 leaves on the crown; trait 501, 1st inflorescence visible; trait 601, 1st flower open; trait 801, 10% fruit fully ripe colour; trait 805, 50% fruit fully ripe colour; and trait 809, 90% fruit fully ripe colour.

We found overall differences for cultivar group (*F*_9, 90_ = 4.763; *p*–value < 0.001), treatment (Pillai’s trace *F*_27, 276_ = 12.228; *p*–value < 0.001), and their interaction (*F*_27, 276_ = 2.155; *p*–value = 0.001). Plants grown in the open field were significantly different from the ones grown in the greenhouse (Table 7 and S1 Table). Cultivar group had significant effects on all studied variables except for the time when the 15^th^ leaf on the stem became unfolded and the time when at least there were 25 unfolded leaves on the crown (Table 7). The interaction between cultivar group × treatment was significant for the times when 10% and 50% of the fruit showed typical fully ripe colour (Table 7).

**Table 7:** Results from the multivariate ANOVA analysis of nine phenological traits.

Factor	MS	DF	*F*-value	*p*-value
**Cultivar group**				
**30% of final length of stem (303)**	**2968.24**	**1, 98**	**9.665**	**0.002**
**1**^**st**^ **primary apical side shoot (201)**	**1171.23**	**1, 98**	**13.359**	**< 0.001**
15^th^ leaf on stem unfolded (115a)	0.42	1, 98	0.001	0.974
25 leaves on the crown (125b)	396.94	1, 98	1.681	0.198
**1st inflorescence visible (501)**	**436.15**	**1, 98**	**6.202**	**0.014**
**1**^**st**^ **flower open (601)**	**842.35**	**1, 98**	**4.355**	**0.039**
**10% fruit ripe colour (801)**	**3789.19**	**1, 98**	**9.052**	**0.003**
**50% fruit ripe colour (805)**	**964.81**	**1, 98**	**15.182**	**< 0.001**
**90% fruit ripe colour (809)**	**600.27**	**1, 98**	**12.341**	**0.001**
**Treatment**				
**30% of final length of stem (303)**	**13368.14**	**3, 98**	**43.527**	**< 0.001**
**1**^**st**^ **primary apical side shoot (201)**	**3091.12**	**3, 98**	**35.256**	**< 0.001**
**15**^**th**^ **leaf on stem unfolded (115a)**	**11705.35**	**3, 98**	**29.018**	**< 0.001**
**25 leaves on the crown (125b)**	**13613.91**	**3, 98**	**57.638**	**< 0.001**
**1st inflorescence visible (501)**	**1167.17**	**3, 98**	**16.596**	**< 0.001**
**1**^**st**^ **flower open (601)**	**6753.22**	**3, 98**	**34.917**	**< 0.001**
**10% fruit ripe colour (801)**	**35549.17**	**3, 98**	**84.925**	**< 0.001**
**50% fruit ripe colour (805)**	**63085.04**	**3, 98**	**992.718**	**< 0.001**
**90% fruit ripe colour (809)**	**77344.57**	**3, 98**	**1590.174**	**< 0.001**
**Treatment × cultivar group**				
30% of final length of stem (303)	174.80	3, 98	0.569	0.637
1^st^ primary apical side shoot (201)	101.36	3, 98	1.156	0.331
15^th^ leaf on stem unfolded (115a)	371.57	3, 98	0.921	0.434
25 leaves on the crown (125b)	516.31	3, 98	2.186	0.095
1st inflorescence visible (501)	44.26	3, 98	0.629	0.598
1^st^ flower open (601)	51.64	3, 98	0.267	0.849
**10% fruit ripe colour (801)**	**1468.99**	**3, 98**	**3.509**	**0.018**
**50% fruit ripe colour (805)**	**219.69**	**3, 98**	**3.457**	**0.019**
90% fruit ripe colour (809)	37.33	3, 98	0.768	0.515

Values in bold are significant.

## Discussion

We have found that variation in environmental conditions related to climate change has dramatic consequences on different traits of the tree tomato crop. Moreover, we found that different crop managements under this new climate conditions produced important differences either on plant architecture and phenology or on crop yield. Overall, traditional tree tomato crop practices are the less productive, and in a warmer scenario the organic fertilization practices are the most favourable to the plants and the farmers. Changes observed in tree tomato phenology as a consequence of treatments simulating new climatic conditions (*i*.*e*., greenhouse cultivation) were expected. Predictions on the effects of climate change on crops, however, point to dramatic yield losses due to the increased frequency of extreme events (e.g. some parts of Europe suffered an increase of above 6°C over long-term means during the heat wave of the summer of 2003) and their impact on plant developmental stages [26]. Thus, temperature plays a key role in determining the yield and quality of fruit, although its interaction with other factors such as irrigation (rainfall) are critical for both the vegetative and reproductive phases of crops [27, 28]. The raising of the temperature and the decrease of the relative humidity had statistically significant positive effects on stem diameter, crown maximum radius, fruit diameter and apex angle, the number of inflorescences and fruit, and yield; and shortened the time to reach the 30% of final length of stem, to produce the 1st primary apical side shoot, and to see both the 15th leaf on stem unfolded and the 1st inflorescence visible. In its natural conditions, *S*. *betaceum* produces larger stems and more nodes, the fruit is longer, and all other phenological traits developed sooner than in the greenhouse conditions.

Therefore, phenology is under strong environmental control and temperature fluctuation drives vegetative flushes (e.g. in mango) with production of more leaves at higher temperatures, which is linked to soil temperature, but preventing or delaying flowering [27]. In other tropical crops, such as sugarcane, the increment of temperature favours yield while there is not water deficit, although predictions point to a drop in yield due to low prices and high labour costs [29]. In other Poaceae crops such as wheat has been observed that water deficit (draught stress) and high temperature, while accelerating tillering, has a negative impact in leaf area, and growth rate [28]. Tree tomato phenological variation not only facilitates its establishment at northern latitudes but renders this species as plastic against climate change effects.

When considering plant architecture, the orange pointed cultivar group reached longer stem length than the purple cultivar group. This measure, however, contrasts with previous findings that were obtained in open field conditions [13]. When comparing the stem length values for orange pointed and purple trees from our open field conditions, the results are as expected; that is, in greenhouse conditions, stem lengths for both cultivar groups are the opposite than in open field conditions. In addition, differences in stem length and the number of nodes might be due to the management between the greenhouse and the traditional open field practices because in the greenhouse tree tomato plants were pruned. Possibly, the latter, combined with the application of different fertilization treatments, could explain the differences in stem diameter, which grows thicker under organic fertilization conditions. Noteworthy, however, is that in other crops moderate temperatures such as the ones in the open field treatment have been found to promote stem length and flowering while higher temperatures promote stem emergence but reduce stem length [30]. We found that the tree crown had its largest expansion in the mineral fertilization treatment, although it did not translate in higher values of fruit characteristics or yield. Plants in the mineral fertilization treatment produced less leaves in the early stages than plants from other treatments.

Overall, fruit characteristics showed the smallest variations among treatments (up to 7.75%). While fruit in the open field tended to have larger lengths, greenhouse conditions seemed to promote larger diameters that are related to fertilization treatment (organic fertilization produced the thicker fruit). The latter is reflected in both the fruit angle and the shape factor. Shape factor is an interesting characteristic because is related to the treatment but the producer might be interested in particular shapes for storage, transportation, or even aesthetical purposes. The length of the pedicel is a trait that varies between the two studied tree tomato cultivar groups but suffers no variation under different treatments, as found previously [13]. That is, pedicel length is a good defining character for these two cultivar groups. The number of inflorescences was lower in the open field but the wind regime must play an important role blowing flowers away. As a fact, wind speeds were stronger during the blossoming season (up to 20.9 km h^-1^). This factor was absent in greenhouse conditions. As expected, a lower number of inflorescences resulted in a reduction in the number of fruit. Although we expected to find larger fruit in the greenhouse conditions, fruit weight was higher in the open field. A potential explanation for this observation is that plant resources, ready to be allocated to flower and fruit development, were invested in the few resistant flowers that survived in the field, while under the greenhouse conditions that provide shelter against the wind more flowers survived and produced more fruit, which means higher demand of water and nutrients, thus, suggesting potential trade-offs in resource allocation analogous to positional decline in allocation [31]. Moreover, we found that purple cultivar group fruit weighted more than the orange pointed cultivar group fruit, as found previously [13]. The number of fruit produced in the greenhouse was three times higher than in open field conditions; and, for cultivar groups, the overall mean number of fruit were around 21, practically as found in a previous work [22]. Yield was drastically lower in the traditional management compared to the milder conditions in the greenhouse, and the organic fertilization was the most effective treatment boosting yield. A positive trend between climatic temperature and tomato yield has been previously reported in South Africa [32].

In general, we found a delay in the vegetative development of the plants produced under traditional management in the open field, while there was an acceleration in the development of reproductive structures and fruit. The latter was expected because the optimal temperatures for vegetative development in many plant species are higher than for reproductive development [1]. In addition, it has been shown that stresses such as heat and draught induce longer vegetative growth and shorter reproductive phase in wheat [28]. While these results suggest that greenhouse temperature conditions are closer to the optimum temperature of the species because of its accelerated vegetative growth, it might be an impasse for the farmer because reproduction is delayed near 100 days. This might be due to the low night temperature in the field (e.g. [33, 34]). In general, the orange pointed cultivar group was 4–6 days faster than the purple cultivar group. While previous work found that the time needed for 50% of the fruit showing typical fully ripe colour were 520 and 470 days for the orange pointed and the purple cultivar groups, our fruit needed 487 and 492 days, respectively [22].

Although the effects of extreme environmental conditions have overall negative effects on plants of agronomical importance, cautious investigation on single local crop species can provide insight on those species that will be able to prosper and even increase their yield in the new conditions; thus, allowing new food and economic opportunities. The interaction between environmental and social factors is determinant to evaluate the fate of crops dynamics. Finally, the knowledge generated after the evaluation of tree tomato cultivars will contribute to generate resilience against climate change effects.

## Supporting information

S1 Table Post hoc tests for differences among nine phenological variables. (PDF)
