# Supplementary material for: The impact of an extreme climatic disturbance and different fertilization treatments on plant development, phenology, and yield of two cultivar groups of Solanum betaceum Cav
Source: PLoS One. 2017 Dec 29;12(12):e0190316. doi: 10.1371/journal.pone.0190316 (PMC5747456; doi:10.1371/journal.pone.0190316)
Supplement: S1 Table — (PDF) [file pone.0190316.s001.pdf]

S1 Table. Post hoc tests for differences among nine phenological variables.

**Variable**	**Treatment**	Organic	Mineral	Control
303	Mineral	1.000		
	Control	0.564	0.630	
	Open field	< 0.001	< 0.001	< 0.001
201	Mineral	0.165		
	Control	0.993	0.146	
	Open field	< 0.001	< 0.001	< 0.001
115a	Mineral	0.851		
	Control	0.385	0.104	
	Open field	< 0.001	< 0.001	< 0.001
125b	Mineral	0.742		
	Control	0.222	0.770	
	Open field	< 0.001	< 0.001	< 0.001
501	Mineral	0.993		
	Control	0.942	0.989	
	Open field	< 0.001	< 0.001	< 0.001
601	Mineral	0.617		
	Control	0.151	0.764	
	Open field	< 0.001	< 0.001	< 0.001
801	Mineral	< 0.001		
	Control	< 0.001	0.337	
	Open field	< 0.001	< 0.001	< 0.001
805	Mineral	0.994		
	Control	0.082	0.155	
	Open field	< 0.001	< 0.001	< 0.001
809	Mineral	0.135		
	Control	0.962	0.458	
	Open field	< 0.001	< 0.001	< 0.001
